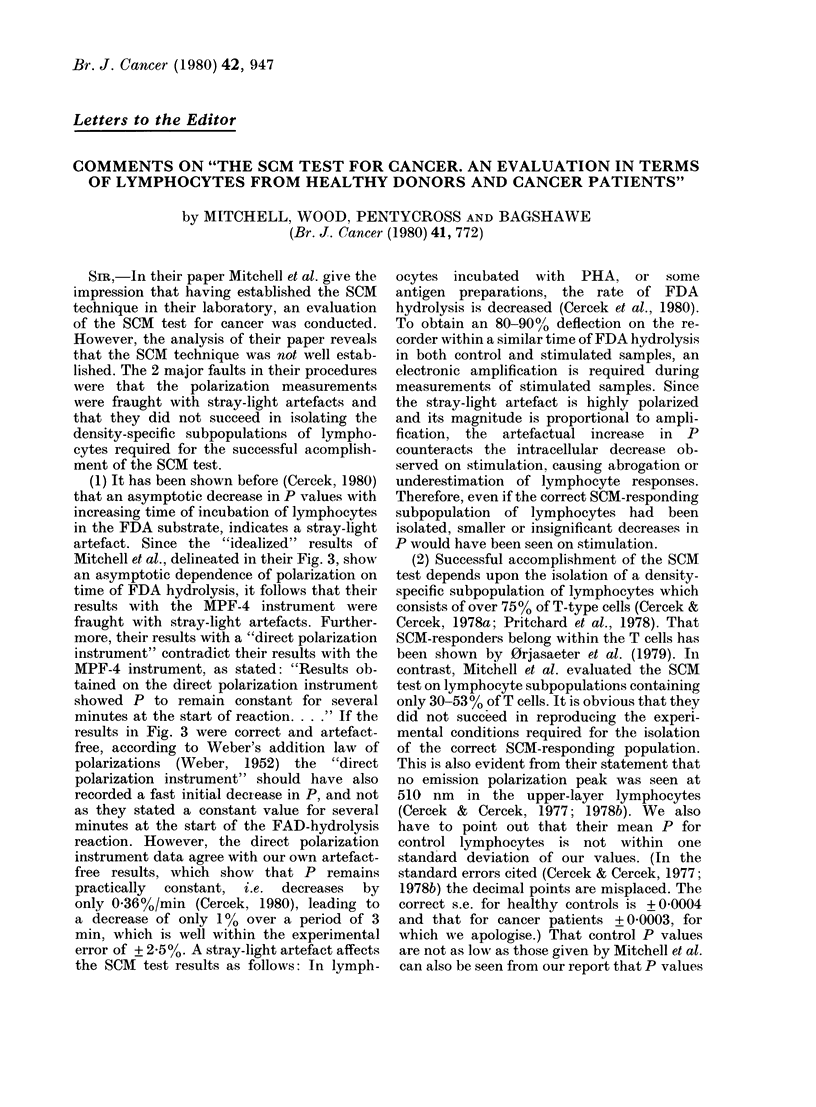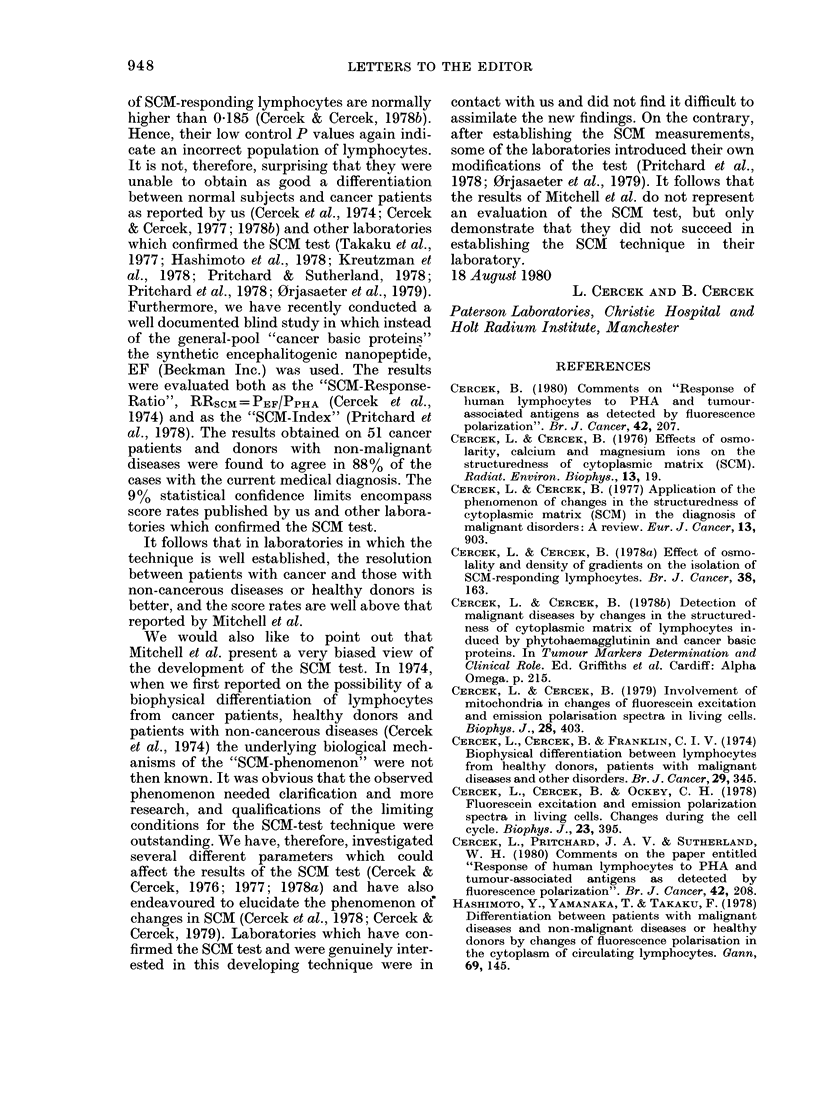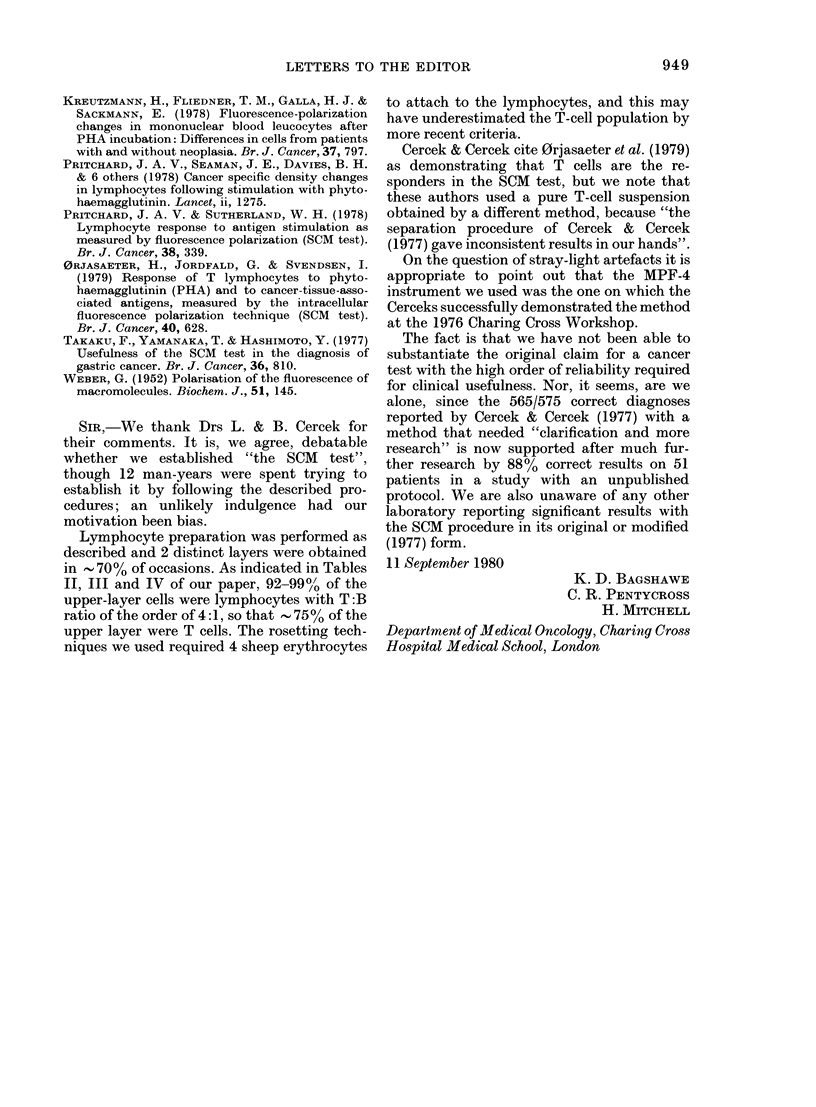# Comments on "the SCM test for cancer. An evaluation in terms of lymphocytes from healthy donors and cancer patients".

**DOI:** 10.1038/bjc.1980.346

**Published:** 1980-12

**Authors:** L. Cercek, B. Cercek


*Br. J. Cancer* (1980) **42**, 947

### *Letters to the Editor*

### COMMENTS ON "THE SCM TEST FOR CANCER. AN EVALUATION IN TERMS OF LYMPHOCYTES FROM HEALTHY DONORS AND CANCER PATIENTS"

by MITCHELL, WOOD, PENTYCROSS AND BAGSHAWE
(*Br. J. Cancer* (1980) **41**, 772)

SIR,—In their paper Mitchell *et al.* give the impression that having established the SCM technique in their laboratory, an evaluation of the SCM test for cancer was conducted. However, the analysis of their paper reveals that the SCM technique was *not* well established. The 2 major faults in their procedures were that the polarization measurements were fraught with stray-light artefacts and that they did not succeed in isolating the density-specific subpopulations of lymphocytes required for the successful acomplishment of the SCM test.

(1) It has been shown before (Cercek, 1980) that an asymptotic decrease in *P* values with increasing time of incubation of lymphocytes in the FDA substrate, indicates a stray-light artefact. Since the "idealized" results of Mitchell *et al.*, delineated in their Fig. 3, show an asymptotic dependence of polarization on time of FDA hydrolysis, it follows that their results with the MPF-4 instrument were fraught with stray-light artefacts. Furthermore, their results with a "direct polarization instrument" contradict their results with the MPF-4 instrument, as stated: "Results obtained on the direct polarization instrument showed *P* to remain constant for several minutes at the start of reaction. . . ." If the results in Fig. 3 were correct and artefact-free, according to Weber's addition law of polarizations (Weber, 1952) the "direct polarization instrument" should have also recorded a fast initial decrease in *P*, and not as they stated a constant value for several minutes at the start of the FAD-hydrolysis reaction. However, the direct polarization instrument data agree with our own artefact-free results, which show that *P* remains practically constant, *i.e.* decreases by only 0·36%/min (Cercek, 1980), leading to a decrease of only 1% over a period of 3 min, which is well within the experimental error of $\pm 2{\cdot}5\%$. A stray-light artefact affects the SCM test results as follows: In lymphocytes incubated with PHA, or some antigen preparations, the rate of FDA hydrolysis is decreased (Cercek *et al.*, 1980). To obtain an 80–90% deflection on the recorder within a similar time of FDA hydrolysis in both control and stimulated samples, an electronic amplification is required during measurements of stimulated samples. Since the stray-light artefact is highly polarized and its magnitude is proportional to amplification, the artefactual increase in *P* counteracts the intracellular decrease observed on stimulation, causing abrogation or underestimation of lymphocyte responses. Therefore, even if the correct SCM-responding subpopulation of lymphocytes had been isolated, smaller or insignificant decreases in *P* would have been seen on stimulation.

(2) Successful accomplishment of the SCM test depends upon the isolation of a density-specific subpopulation of lymphocytes which consists of over 75% of T-type cells (Cercek & Cercek, 1978*a*; Pritchard *et al.*, 1978). That SCM-responders belong within the T cells has been shown by Ørjasaeter *et al.* (1979). In contrast, Mitchell *et al.* evaluated the SCM test on lymphocyte subpopulations containing only 30–53% of T cells. It is obvious that they did not succeed in reproducing the experimental conditions required for the isolation of the correct SCM-responding population. This is also evident from their statement that no emission polarization peak was seen at 510 nm in the upper-layer lymphocytes (Cercek & Cercek, 1977; 1978*b*). We also have to point out that their mean *P* for control lymphocytes is not within one standard deviation of our values. (In the standard errors cited (Cercek & Cercek, 1977; 1978*b*) the decimal points are misplaced. The correct s.e. for healthy controls is $\pm 0{\cdot}0004$ and that for cancer patients $\pm 0{\cdot}0003$, for which we apologise.) That control *P* values are not as low as those given by Mitchell *et al.* can also be seen from our report that *P* values of SCM-responding lymphocytes are normally higher than 0·185 (Cercek & Cercek, 1978*b*). Hence, their low control $P$ values again indicate an incorrect population of lymphocytes. It is not, therefore, surprising that they were unable to obtain as good a differentiation between normal subjects and cancer patients as reported by us (Cercek *et al.*, 1974; Cercek & Cercek, 1977; 1978*b*) and other laboratories which confirmed the SCM test (Takaku *et al.*, 1977; Hashimoto *et al.*, 1978; Kreutzman *et al.*, 1978; Pritchard & Sutherland, 1978; Pritchard *et al.*, 1978; Ørjasaeter *et al.*, 1979). Furthermore, we have recently conducted a well documented blind study in which instead of the general-pool "cancer basic proteins" the synthetic encephalitogenic nanopeptide, EF (Beckman Inc.) was used. The results were evaluated both as the "SCM-Response-Ratio", $RR_{SCM} = P_{EF}/P_{PHA}$ (Cercek *et al.*, 1974) and as the "SCM-Index" (Pritchard *et al.*, 1978). The results obtained on 51 cancer patients and donors with non-malignant diseases were found to agree in 88% of the cases with the current medical diagnosis. The 9% statistical confidence limits encompass score rates published by us and other laboratories which confirmed the SCM test.

It follows that in laboratories in which the technique is well established, the resolution between patients with cancer and those with non-cancerous diseases or healthy donors is better, and the score rates are well above that reported by Mitchell *et al.*

We would also like to point out that Mitchell *et al.* present a very biased view of the development of the SCM test. In 1974, when we first reported on the possibility of a biophysical differentiation of lymphocytes from cancer patients, healthy donors and patients with non-cancerous diseases (Cercek *et al.*, 1974) the underlying biological mechanisms of the "SCM-phenomenon" were not then known. It was obvious that the observed phenomenon needed clarification and more research, and qualifications of the limiting conditions for the SCM-test technique were outstanding. We have, therefore, investigated several different parameters which could affect the results of the SCM test (Cercek & Cercek, 1976; 1977; 1978*a*) and have also endeavoured to elucidate the phenomenon of changes in SCM (Cercek *et al.*, 1978; Cercek & Cercek, 1979). Laboratories which have confirmed the SCM test and were genuinely interested in this developing technique were in contact with us and did not find it difficult to assimilate the new findings. On the contrary, after establishing the SCM measurements, some of the laboratories introduced their own modifications of the test (Pritchard *et al.*, 1978; Ørjasaeter *et al.*, 1979). It follows that the results of Mitchell *et al.* do not represent an evaluation of the SCM test, but only demonstrate that they did not succeed in establishing the SCM technique in their laboratory.

18 *August* 1980

L. Cercek and B. Cercek

*Paterson Laboratories, Christie Hospital and Holt Radium Institute, Manchester*

### REFERENCES

Cercek, B. (1980) Comments on "Response of human lymphocytes to PHA and tumour-associated antigens as detected by fluorescence polarization". *Br. J. Cancer*, **42**, 207.

Cercek, L. & Cercek, B. (1976) Effects of osmolarity, calcium and magnesium ions on the structuredness of cytoplasmic matrix (SCM). *Radiat. Environ. Biophys.*, **13**, 19.

Cercek, L. & Cercek, B. (1977) Application of the phenomenon of changes in the structuredness of cytoplasmic matrix (SCM) in the diagnosis of malignant disorders: A review. *Eur. J. Cancer*, **13**, 903.

Cercek, L. & Cercek, B. (1978*a*) Effect of osmolality and density of gradients on the isolation of SCM-responding lymphocytes. *Br. J. Cancer*, **38**, 163.

Cercek, L. & Cercek, B. (1978*b*) Detection of malignant diseases by changes in the structuredness of cytoplasmic matrix of lymphocytes induced by phytohaemagglutinin and cancer basic proteins. In *Tumour Markers Determination and Clinical Role*. Ed. Griffiths *et al.* Cardiff: Alpha Omega. p. 215.

Cercek, L. & Cercek, B. (1979) Involvement of mitochondria in changes of fluorescein excitation and emission polarisation spectra in living cells. *Biophys. J.*, **28**, 403.

Cercek, L., Cercek, B. & Franklin, C. I. V. (1974) Biophysical differentiation between lymphocytes from healthy donors, patients with malignant diseases and other disorders. *Br. J. Cancer*, **29**, 345.

Cercek, L., Cercek, B. & Ockey, C. H. (1978) Fluorescein excitation and emission polarization spectra in living cells. Changes during the cell cycle. *Biophys. J.*, **23**, 395.

Cercek, L., Pritchard, J. A. V. & Sutherland, W. H. (1980) Comments on the paper entitled "Response of human lymphocytes to PHA and tumour-associated antigens as detected by fluorescence polarization". *Br. J. Cancer*, **42**, 208.

Hashimoto, Y., Yamanaka, T. & Takaku, F. (1978) Differentiation between patients with malignant diseases and non-malignant diseases or healthy donors by changes of fluorescence polarisation in the cytoplasm of circulating lymphocytes. *Gann*, **69**, 145.

Kreutzmann, H., Fliedner, T. M., Galla, H. J. & Sackmann, E. (1978) Fluorescence-polarization changes in mononuclear blood leucocytes after PHA incubation: Differences in cells from patients with and without neoplasia. *Br. J. Cancer*, **37**, 797.

Pritchard, J. A. V., Seaman, J. E., Davies, B. H. & 6 others (1978) Cancer specific density changes in lymphocytes following stimulation with phytohaemagglutinin. *Lancet*, ii, 1275.

Pritchard, J. A. V. & Sutherland, W. H. (1978) Lymphocyte response to antigen stimulation as measured by fluorescence polarization (SCM test). *Br. J. Cancer*, **38**, 339.

Ørjasaeter, H., Jordfald, G. & Svendsen, I. (1979) Response of T lymphocytes to phytohaemagglutinin (PHA) and to cancer-tissue-associated antigens, measured by the intracellular fluorescence polarization technique (SCM test). *Br. J. Cancer*, **40**, 628.

Takaku, F., Yamanaka, T. & Hashimoto, Y. (1977) Usefulness of the SCM test in the diagnosis of gastric cancer. *Br. J. Cancer*, **36**, 810.

Weber, G. (1952) Polarisation of the fluorescence of macromolecules. *Biochem. J.*, **51**, 145.